# Comparison of femoral neck system vs. dynamic hip system blade for the treatment of femoral neck fracture in young patients: A retrospective study

**DOI:** 10.3389/fsurg.2023.1092786

**Published:** 2023-02-03

**Authors:** Zilu Ge, Wei Xiong, Dong Wang, Yunfeng Tang, Qian Fang, Limin Wang, Zhen Zhang, Wei Lin, Guanglin Wang

**Affiliations:** ^1^ Department of Orthopedics, Orthopedic Research Institute, West China Hospital, Sichuan University, Chengdu, China; ^2^ West China Women's and Children's Hospital, Sichuan University, Chengdu, China

**Keywords:** femoral neck system, dynamic hip system blade, femoral neck fracture, clinical outcomes, young

## Abstract

**Background:**

Femoral neck fracture is a common fracture in orthopedic practice. This study aimed to compare the clinical outcomes between the femoral neck system and dynamic hip system blade for the treatment of femoral neck fracture in young patients.

**Methods:**

This retrospective study included 43 and 52 patients who underwent treatment for femoral neck fracture with the femoral neck system and dynamic hip system blade, respectively, between August 2019 and August 2020. Operative indexes, including operation duration, blood loss, incision length, postoperative complications (femoral neck shortening, non-union, screw pull-out, femoral head necrosis), and Harris scale scores were recorded and analyzed.

**Results:**

Compared to that with the dynamic hip system blade, the femoral neck system showed significantly less operation duration (femoral neck system vs. dynamic hip system blade: 47.09 ± 9.19 vs. 52.90 ± 9.64, *P* = 0.004), less blood loss (48.53 ± 10.69 vs. 65.31 ± 17.91, *P* < 0.001), and shorter incision length (4.04 ± 0.43 vs. 4.93 ± 0.53, *P* < 0.001). Femoral neck shortening was significantly lower with the femoral neck system than with the dynamic hip system blade (3.93 ± 2.40, *n* = 39 vs. 5.22 ± 2.89, *n* = 44, *P* = 0.031). No statistical differences were observed between the two groups in nonunion, screw pull-out, and femoral head necrosis. In addition, the latest follow-up Harris scale score was significantly higher with the femoral neck system than with the dynamic hip system blade (92.3 ± 4.5 vs. 89. 9 ± 4.9, *P* = 0.015).

**Conclusion:**

The femoral neck system results in less trauma, less femoral neck shortening, and better hip joint function than the dynamic hip system blade for the treatment of femoral neck fracture in young patients.

## 1. Introduction

Femoral neck fracture (FNF) is one of the most common types of fracture in orthopedic practice (1). Although the incidence of FNF is relatively lower in younger patients than in older patients, the occurrence of FNF normally results from high-energy trauma in young adult patients, often represented by displaced and unstable fracture patterns (2). Currently, the most common types of fixation include cannulated screws, hip screw systems, proximal femur plates, and cephalomedullary nails (3). Arthroplasty may be an option for elderly patients, but is generally not feasible for young patients; young patients with FNF require a more durable and promising fixation. However, there is no consensus on the best fixation method for FNF in young patients (4).

The dynamic hip screw (DHS), first introduced by Clawson in 1964, has been widely used in the treatment of FNF (5). Based on the mechanical properties of the DHS, a DHS blade (DHSb), with extra helical blades, was developed to enhance the anchorage ability of the fixation to the bone. Studies have shown that DHSb can achieve satisfactory biomechanical properties and clinical outcomes in patients with FNF (6, 7).

The femoral neck system (FNS) is a new fixation device, consisting of a locking plate, neck bolt, and antirotation screw. In this system, the neck bolt provides angular stability, the antirotation screw provides rotational stability, and the locking plate resists torsional force. A biomechanical evaluation of human cadaveric femora supported the FNS as an effective alternative to DHS and cannulated screws for the treatment of FNF (8). Another clinical study reported that patients with FNF who received FNS treatment had better clinical outcomes than those treated with cannulated compression screws (9). However, to our knowledge, no study has compared the clinical outcomes of FNS and DHSb in young patients with FNF.

Therefore, this retrospective study aimed to compare the clinical outcomes of FNS and DHSb, with perioperative characteristics, hip function, and postoperative complications as the primary outcomes, and determine the more effective fixation method for young patients with FNF.

### 1.1. Patients and methods

Records of patients with FNF who underwent FNS or DHSb from August 2019 to August 2020 at the Department of Orthopedics, West China Hospital, Sichuan University (Sichuan, China) were retrospectively reviewed. This study was approved by the Clinical Academic Committee of West China Hospital (No. 2021132), and was conducted in compliance with the Helsinki Declaration. Informed consent was obtained from all study subjects.

Specific inclusion and exclusion criteria were set for all patients. The inclusion criteria were as follows: (1) aged between 18 and 65 years; (2) underwent unilateral primary FNF surgery; (3) related follow-up records, including radiography and Harris scale evaluation, were comprehensive; and (4) a minimum postoperative follow-up of 18 months. The exclusion criteria were as follows: (1) pathological fractures or open fractures; and (2) local infection in the hip joint before the fixation surgery. According to the inclusion and exclusion criteria, 95 patients were finally included in the present study. Patients were divided into FNS (*n* = 43) and DHSb groups (*n* = 52). Data regarding age, sex, body mass index (BMI), smoking, operative side, follow-up duration, and Garden and Pauwels type were collected.

### 1.2. Operative techniques

#### 1.2.1. Femoral neck system

All surgeries were performed by qualified surgeons. Under general or epidural anesthesia, patients were placed in an orthopedic traction device in the supine position. Open reduction was performed if the reduction did not meet the I and II levels under C-arm fluoroscopy. After a 4-cm longitudinal incision below the greater trochanter was performed, a temporary wire was used as an antirotation wire in the superior/anterior portion of the femoral neck to prevent inadvertent rotation of the femoral head. A second wire was then inserted using a 130°-angled guide. After ensuring that the wire was central to the femoral neck and head by C-arm fluoroscopy, a measuring device was used to determine the length. The implant (Depuy Synthes, USA) was inserted over the central guidewire into the pre-reamed hole. The locking screw and antirotation screw were then inserted into the implant under C-arm fluoroscopy. When fixation was confirmed after the final tightening, the subcutaneous tissue was repaired with a 3-0 absorbable suture, and the skin was closed with a 3-0 nonabsorbable suture.

#### 1.2.2. Dynamic hip system blade

**Figure 1:**
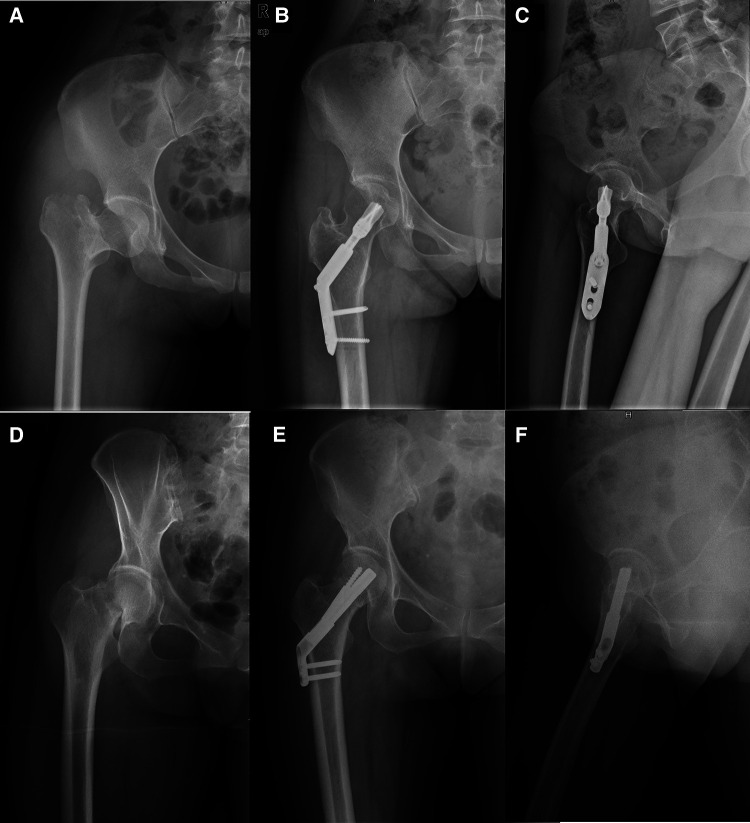
Two internal fixation methods for femoral neck fracture. A 48-year-old female received Dynamic hip system blade fixation after 20 months (**A–C**) and A 50-year-old female received femoral neck system after 19 months (**D–F**).

The process of anesthesia and reduction was the same as that described above. A similar, but longer, full-thickness incision was performed. After a guide pin was inserted under C-arm fluoroscopy, the femoral shaft was reamed along the direction of the guide pin. The DHSb was inserted into the femoral head, and the tip was positioned approximately 5–10 mm beneath the surface of the femoral cartilage. The side plate was fixed close to the bone surface and locked with two or three locking screws. Finally, the screw caps and blade were tightened. The wound was then washed and sutured (Figure 1).

#### 1.2.3. Postoperative management

Both groups received the same postoperative management. Antibiotics were administered 0.5 h before, and 24 h after, the surgery. After anesthesia, patients were taught and encouraged to perform active isometric contraction of the lower limb muscles, active ankle motion, and passive hip joint motion. For stable fractures, patients were allowed partial weight-bearing. Based on the patient's weight, 0.2–0.4 ml low molecular heparin was used during hospitalization. On discharge, patients received loxoprofen sodium (180 mg/day) and rivaroxaban (10 mg/day) for three weeks to prevent pain and the occurrence of deep venous thrombosis. At 6 weeks to 3 months after surgery, partial weight-bearing with a cane was allowed. After 3 months, patients were allowed full weight-bearing based on x-ray examination findings.

#### 1.2.4. Clinical evaluations

Related clinical data were retrieved from the hospital database. All patients were required to undergo x-ray examination at 6 weeks, 3 months, 6 months, 1 year postoperatively and last follow-up. Preoperative baseline characteristics, surgical information, and postoperative Harris scale score (10) were collected by medical staff blinded to this study. Patients were required to return to the hospital for a final evaluation. Two qualified doctors blinded to this study was in charge of the last follow-up evaluation in March 2022. The Harris scale score was used to evaluate hip joint function. And the points were graded as follows: 90–100 was excellent, 80–89 was good, 70–79 was medium and less than 70 was poor. Femoral head necrosis was assessed according to the standard of Slobogean et al. (11). Femoral neck shortening based on x-ray examination was recorded and categorized as non/mild (<5 mm), moderate (5–10 mm), or severe (>10 mm) (12, 13).

#### 1.2.5. Statistical analysis

All data were analyzed using SPSS (version 22.0; IBM, Chicago, IL, USA). Continuous variables are reported as the mean ± standard deviation. Discrete variables are reported as numbers (percentages). The student's t-test or paired-samples t-test was used to compare continuous variables. The chi-squared test or Fisher's exact test was used to compare categorical data. Statistical significance was set at *P* < 0.05.

## 2. Results

The study population comprised 95 patients, including 43 and 52 patients in the FNS and DHSb groups, respectively. The average follow-up period was 24.3 ± 3.1 months in the DHSb group and 25.8 ± 4.2 months in the FNS group (*P* = 0.274). Patient demographics, including age, sex, BMI, follow-up, operative side, and smoking not significantly different between the two groups. Most fractures were III-IV Garden type or II-III Pauwels type, with no significant difference in fracture type between the two groups. Background data are summarized in Table 1.

**Table 1:** Summary of patients’ demographics.

	DHSb group (*n* = 52)	FNS group (*n* = 43)	*P* value
Age			0.157
Mean ± SD	48.2 ± 8.0	50.4 ± 7.4	
Median	48.0	51.0	
Range (min-max)	30-65	35-64	
Gender			0.883
Female, *n* (%)	15 (28.8)	13 (30.2)	
Male *n* (%)	37 (71.2)	30 (69.8)	
BMI			0.292
Mean ± SD	22.1 ± 2.4	22.2 ± 2.3	
Median	22.2	22.1	
Range (min-max)	17.8–27.4	18.8–27.9	
Follow-up (months)			0.274
Mean ± SD	24.3 ± 3.1	25.8 ± 4.2	
Median	23.9	24.8	
Range (min-max)	19–30	19–30	
Operative Side n (%)			0.056
Left	20 (38.5)	25 (58.1)	
Right	32 (61.5)	18 (41.9)	
Smoking *n* (%)			0.410
Smoker	12 (23.1)	7 (16.3)	
Non-smoker	40 (76.9)	36 (83.7)	
Garden Type *n* (%)			0.897
Type II	7 (13.4)	5 (11.6)	
Type III	29 (55.8)	26 (60.5)	
Type IV	16 (30.8)	12 (27.9)	
Pauwels Type *n* (%)			0.748
Type I	8 (15.4)	5 (11.6)	
Type II	14 (26.9)	10 (23.3)	
Type III	30 (57.7)	28 (65.1)	
Open reduction *n* (%)			0.843
Open	13 (25.0)	10 (23.3)	
Closed	39 (75.0)	33 (76.7)	

Surgical information, including operation duration, blood loss, incision length, hospitalization time, and x-ray time during surgery, were recorded and analyzed (Table 2). Compared to that in the DHSb group, the FNS group had significantly less operation duration (FNS vs. DHSb: 47.09 ± 9.19 vs. 52.90 ± 9.64, *P* = 0.004), less blood loss (48.53 ± 10.69 vs. 65.31 ± 17.91, *P* < 0.001), and shorter incision length (4.04 ± 0.43 vs. 4.93 ± 0.53, *P* < 0.001). There was no statistically significant difference in hospitalization time between the two groups.

**Table 2:** Operation information of both groups.

	DHSb group (*n* = 52)	FNS group (*n* = 43)	*P* value
Operation duration (min)	52.90 ± 9.64	47.09 ± 9.19	0.004^a^
Blood loss (ml)	65.31 ± 17.91	48.53 ± 10.69	<0.001^a^
Incision length (cm)	4.93 ± 0.53	4.04 ± 0.43	<0.001^a^
Hospitalization time (day)	4.94 ± 0.89	5.05 ± 1.00	0.593

^a^ There had statistical difference.

Postoperative complications, including femoral neck shortening, nonunion, screw pull-out, femoral head necrosis, and infection, were recorded and compared (Table 3). In both groups, most patients had satisfactory outcomes. Femoral neck shortening was significantly less in the FNS group than in the DHSb group (3.93 ± 2.40, *n* = 39 vs. 5.22 ± 2.89, *n* = 44, *P* = 0.031). The percentage of patients with femoral neck shortening of <10 mm at the last follow-up was 78.8% in the DHSb group and 86.0% in the FNS group. However, three cases in the DHSb group and two cases in the FNS group had femoral neck shortening of >10 mm postoperatively. Although the number of cases of severe clinical outcomes, including non-union, screw pull-out, and femoral head necrosis, was lower in the FNS group than in the DHSb group, there was no statistical difference between the two groups (*P* = 0.537). There was one case in the DHSb group of *Staphylococcus aureus* infection at the incision region at 5 days postoperatively. Based on the bacterial culturing results and drug sensitivity tests, cefazolin was used. The incision healed well, and no signs of infection were observed around the internal fixation and bone fracture. No further debridement was performed.

**Table 3:** Postoperative complications and Harris scale score for both groups.

	DHSb group (*n* = 52)	FNS group (*n* = 43)	*P* value
Femoral neck shortening (mm)	5.22 ± 2.89 (*n* = 44)	3.93 ± 2.40 (*n* = 39)	0.031^a^
<5 mm (*n*, %)	29 (55.8)	30 (69.8)	
5–10 mm (*n*, %)	12 (23.0)	7 (16.2)	
>10 mm (*n*, %)	3 (5.8)	2 (4.7)	
Total (*n*, %)	8 (15.4)	4 (9.3)	0.537
Nonunion	3 (5.8)	2 (4.7)	
Screw pull-out	2 (3.8)	1 (2.3)	
Femoral head necrosis	3 (5.8)	1 (2.3)	
Harris scale	89. 9 ± 4.9	92.3 ± 4.5	0.015^a^

^a^ There had statistical difference.

Data on hip joint function as assessed by Harris scale are presented in Table 3. There were 5 patients had poor, 8 had medium, 28 had good and 11 had excellent Harris scores in DNSb group. And there were 3 patients had poor, 5 had medium, 20 had good and 15 had excellent scores in FNS group. The FNS group showed statistically better hip joint function than the DHSb group at the last follow-up (92.3 ± 4.5 vs. 89. 9 ± 4.9, *P* = 0.015).

## 3. Discussion

The most important finding of this study was that the FNS resulted in less trauma and better hip joint function at the last follow-up than the DHSb for the treatment of FNF in young patients. In addition, femoral neck shortening was lower in the FNS group than in the DHSb group. Other postoperative complications, including nonunion, screw pull-out, and femoral head necrosis, showed no statistical difference between the two groups.

In the present study, the FNS group had significantly shorter operation duration, less blood loss, and shorter incision length than the DHSb group. Thus, FNS resulted in less trauma than DHSb. Because of its smaller plate, with a compact design and customized operation device, the FNS had a reduced implant footprint on the bone. In addition, the bolt design could control the femoral head depth and thus avoid protrusion; lateral protrusion can result in thigh pain, which can reflect a theoretical remission. Furthermore, less insertional torque was produced during insertion. These advantages contributed to less intraoperative x-ray irradiation and simplified operative processes, reducing the operation duration and complication occurrence. However, use of this newly designed device incurred a higher cost, leading to a more expensive hospitalization charge in the FNS group than in the DHSb group.

FNF treatment in young patients is focused on five key aspects, including fracture reduction maintenance, femoral neck shortening prevention, femoral head necrosis prevention, better healing promotion, and FNF prevention (14). Among these, a durable and rigid internal fixation is the primary factor in treating the fracture. Young patients normally have a high hip joint function demand, but are not ideal candidates for arthroplasty. Although young patients have a better blood supply and potential healing ability than older patients, appropriate fixation methods are still essential to promote better healing. As with many other medical conditions, the treatment must be adapted to the unique features of this population. Accordingly, effective internal fixation can delay and even avoid arthroplasty in young patients.

Various internal fixation methods have been compared in several recent studies. A finite element analysis showed that both cannulated screws and DHS could resist shearing and rotational forces (15). Kuan et al. suggested that compression hip screw fixation was superior to both the modified cross-screw fixation method and the inverted triangle fixation method for Pauwels III FNFs (16). The DHSb has been reported to have better resistance to pushout and rotational stability compared to that with dynamic hip screw in a biomechanical cadaveric test (6). Another study showed that DHS combined with fibula bone had better clinical outcomes than cannulated screws in Pauwels type III FNF (17). However, Stoffel et al. demonstrated that the FNS had greater axial stiffness than the DHSb and cannulated screws based on 20 pairs of cadaveric femora (8). The outcomes of the finite element and biomechanical analyses are not exactly consistent, and a clinical trial is still needed.

Some studies have aimed to determine the superior method. In a study with an average follow-up of 27 months, DHSb showed better clinical outcomes than cannulated compression screws in preventing femoral neck shortening, screw migration, and cut-out; however, there was no significant difference in postoperative fracture union (18). In addition, Hu et al. reported that only the occurrence of femoral neck shortening was significantly less with the FNS than with cannulated compression screws; no statistical difference was observed in femoral head necrosis and fracture nonunion between the two groups (9). However, to date, no clinical trial has investigated the clinical outcomes of FNS and DHSb in young patients. In our study, although both methods achieved satisfactory clinical outcomes, the FNS group had better hip joint function than the DHSb group. This may have resulted from less trauma and better biomechanical properties with the FNS than with the DHSb.

The postoperative complications of FNF have become one of the biggest reasons for the choice of internal fixation. Femoral head necrosis, nonunion, fixation failure, and femoral neck shortening are the most common postoperative complications (19). A previous study reported the non-union rate of Pauwels type-3 vertical FNFs as 19% when treated with calcaneal screws and 8% when treated with a fixed-angle device (20). Stockton et al. reported that 32% of young patients with FNF experienced neck shortening of >1 cm when treated with cancellous screws or sliding hip screws (21). The characteristics of the FNF itself determine the high incidence of complications. A high shear force and varus instability could result in fixation failure and nonunion (22). Cancellous screws can only provide limited resistance to vertical shear forces at the fracture site (23). Comparing with the cancellous screws and DHSb, the FNS was designed for more bone retention and fracture fixation properties increasing. Screw-locking into the bolt may contribute to two fracture components sliding together for dynamic fixation; this design can reduce the occurrence of complications. In our study, 4 patients (9.3%) in the FNS group and 8 patients (15.4%) in DHSb group had non-union, screw pull-out, or femoral head necrosis. Although there was no significant difference between the two groups, these methods still showed better clinical prospects than cancellous screws based on previous data. In addition, femoral neck shortening was significantly better in the FNS group than in the DHSb group, rendering it possible for young patients to have better hip function.

There are some limitations to the present study. First, in this retrospective study, patients were not randomly assigned to the two treatment groups. The physician's preference for FNS or DHSb and the preoperative conversation may influence the patients' psychology, thus affecting their recovery. Therefore, a random, multi-center, prospective study is required to further prove the present outcomes. Second, the average follow-up duration was somewhat short in the present study. The final destination of the femoral head remains to be observed. Third, due to limits of fixed post postoperatively evaluation, we couldn't assess the accurate healing time of patients, which might affect patients' recovery training. Furthermore, although we intended to investigate the superior method for young patients; the average age was still approximately 48–50 years. As the present study is limited by a small sample size for younger patients, the collection of more cases and additional stratification analyses on age could provide more convincing conclusions for the treatment of younger patients.

## 4. Conclusion

The FNS results in less trauma, less femoral neck shortening, and better hip joint function than the DHSb for the treatment of FNF in young patients. Thus, the FNS method may have a promising future in the treatment of younger patients.

## Data Availability

The raw data supporting the conclusions of this article will be made available by the authors, without undue reservation.
